# Lifestyle-related predictors affecting prediabetes and diabetes in 20-30-year-old young Korean adults

**DOI:** 10.4178/epih.e2020014

**Published:** 2020-03-19

**Authors:** Kyong Sil Park, Seon Young Hwang

**Affiliations:** School of Nursing, Hanyang University , Seoul, Korea

**Keywords:** Diabetes, Prediabetes, Lifestyle, Physical inactivity, Sedentary behavior, Young adult

## Abstract

**OBJECTIVES:**

To investigate lifestyle-related predictors of prediabetes and diabetes in young adults aged 20–39 years using data from the 2014-2016 Korea National Health and Nutrition Examination Survey (KNHANES).

**METHODS:**

This study is a cross-sectional, secondary analysis using the KNHANES data. Participants were classified into normal group (fasting plasma glucose [FPG] <100 mg/dL and/or hemoglobin A1c [HbA1c] <5.7%), prediabetes group (FPG 100-125 mg/dL and/or HbA1c 5.7-6.4%), and diabetes group (FPG ≥126 mg/dL and/or HbA1c ≥ 6.5%). The data were statistically analyzed using SPSS software.

**RESULTS:**

Out of 4,190 participants, 27.7% of men and 16.3% of women were in the prediabetes group and 1.4% of men and 1.3% of women were in the diabetes group. Logistic regression confirmed that age and obesity are predictors of prediabetes and diabetes in both men and women. Additionally low physical activity and low education level are predictors of prediabetes in men and women, respectively (p<0.05).

**CONCLUSIONS:**

This study has found that age and increased obesity are predictors of elevated blood glucose in young men and women in their 20s and 30s. A strategy to lower obesity by promoting physical activity in men in their 30s is essential to prevent metabolic syndrome and progression to prediabetes.

## INTRODUCTION

The prevalence of diabetes is continuously increasing worldwide [1] in both men and women with increasing age. Approximately 14.4% of adults aged 30 years or older have diabetes, and the prevalence of prediabetes too is high with 31.0% in men and 19.7% among women. Although type 2 diabetes continues to be most prevalent among adults in their 40s or higher, it cannot be considered an illness that primarily affects middle-aged adults any more, as the prevalence of prediabetes has risen among younger adults recently [2], with the age of onset of types 2 diabetes decreasing worldwide [1]. In particular, among people in their 30s, who are most economically active but most vulnerable to health management, only about 50% of patients with diabetes are aware of it [3], and the prevalence of prediabetes is about five times higher than that of diabetes [2]. Further, health examination rate is also lower among the 30s compared to the other age groups, which hinders accurate monitoring of prevalence [4]. As shown here, awareness and management of diabetes is extremely low among young adults, which calls for particular attention to this age group for primary prevention. In addition, since it was confirmed that hemoglobin A1c (HbA1c) is useful in the diagnosis of prediabetes and diabetes and was added to the diagnostic criteria for diabetes in 2018, both fasting plasma glucose (FPG) and HbA1c should be included in the examination of the prevalence and relevant factors [2].

As physical inactivity is closely associated with diabetes, World Health Organization (WHO) recommends engaging in at least 30 minutes of moderate-intensity physical activity (PA) [1]. According to a systematic review, increasing moderate-intensity PA was found to lower FPG and HbA1c [5]. Recently, sedentary lifestyle has been identified as another lifestyle risk factor [6]. Due to advances in transportation and work environment, sedentary behavioral patterns have become common, particularly in adults from high-income countries who spend a considerable amount of their awake time in sedentary behavior [7]. Several studies have reported that the risk for diabetes and cardiovascular disease (CVD) increases with increased sedentary time [8,9]. However, PA compliance rate among adolescents, which would continue onto young adulthood, decreased [10], and the practice of walking, aerobic, and muscular exercise in adults has decreased [11]. Due to long work hours and availability of ultra-high-speed Internet, there is a high sedentary behavioral pattern in Korea as well [12]. As physical inactivity among young adults, who are academically and economically active and vigorously consume the Internet, may elevate the risk of prediabetes and diabetes in the future, their prevention and management is crucial.

The risk for prediabetes and diabetes increases with increasing body mass index (BMI), which is the most widely utilized index to classify the obesity level [13], in contrast continuous weight loss was found to delay the progression of prediabetes and diabetes [14]. Particularly, frequent eating out and skipping breakfast elevates HbA1c and FPG levels and are closely associated with type 2 diabetes [15,16]. Recently, about 41.6% of adult men and 25.6% of adult women are obese, with the obesity rate the highest at 46.7% among people in their 30s. People in their 30s also demonstrate unhealthy eating habits, including excessive fat, drink, and sodium intake with increased consumption of non-home-cooked and convenience foods [11]. Therefore, it is necessary to examine the impact of the poor diet pattern and overweight on blood glucose in the young adults.

Smoking was found to be closely associated with type 2 diabetes [14,17,18]. Active and passive smoking increased the risk for type 2 diabetes [17], and smokers with type 2 diabetes had a higher risk for microvascular complications and glucose regulation compared to non-smokers [14]. According to a systematic review [18], smokers were at a 38% higher risk while past smokers were at a 19% higher risk for type 2 diabetes compared to non-smokers. Alcohol consumption has a U-shaped relationship with diabetes where high alcohol consumption has been reported as a risk factor for diabetes [19]. As a result of the unique drinking culture in Korea, monthly binge drinking rate is approximately twice as high among men compared to women, where monthly binge drinking rate is higher than 50% among men in their 20s and 30s and around 46% among women in their 20s [11]. As high alcohol consumption can elevate the risk for diabetes in young adults, managing drinking habits early on is critical.

In Korea, studies on young adults generally examined the association between lifestyle and knowledge [20], factors related to self-care of diabetes [21], and clinical features of diabetes [22], as opposed to lifestyle factors in which very few studies have identified manageable lifestyle factors to improve type 2 diabetes. Therefore, the aim of study is to classify young adults into normal, prediabetes, and diabetes groups according to their FPG and HbA1c levels and to identify lifestyle predictors of prediabetes and diabetes by comparing them with the normal group. Ultimately, we attempt to present evidence supporting the importance of lifestyle management in young adults for the primary prevention of diabetes and contribute to promoting a healthy lifestyle among young adults.

## MATERIALS AND METHODS

### Research design

This study is a cross-sectional, secondary analysis using the Korea National Health and Nutrition Examination Survey (KNHANES) data aiming to identify the lifestyle-related predictors of prediabetes and diabetes in young adults in their 20s and 30s.

### Study population

**Figure 1.**
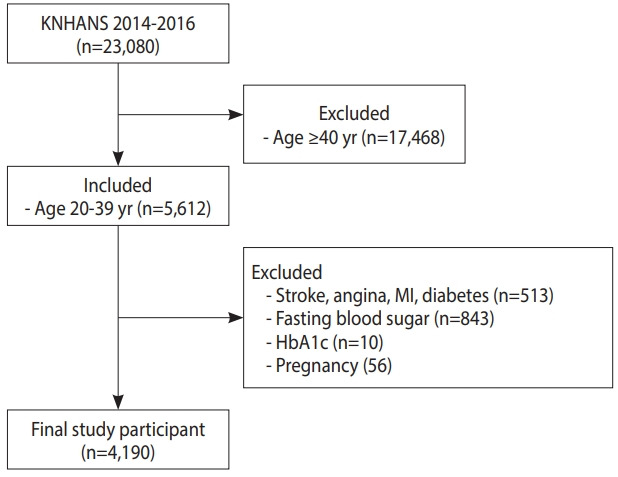
Flow chart of the study sample. KNHANES, Korea National Health and Nutrition Examination Survey; MI, myocardial infarction; HbA1c, hemoglobin A1c.

Participants were selected from the 2014-2016 KNHANES raw data. Among 23,080 participants in the survey, 5,612 people who meet the age criterion of 20-39 years were screened. After excluding 513 subjects who had illnesses such stroke, angina, myocardial infarction, and diabetes during the time of this study based on diagnosis by a physician or use of relevant medications, 5,099 participants were selected. After additionally excluding 843 with data missing for FPG, 10 with data missing for HbA1c, and 56 pregnant women, finally 4,190 participants were enrolled in this study (Figure 1).

The participants were divided into normal, prediabetes, and diabetes groups based on the FPG and HbA1c levels. Normal group was defined as a FPG < 100 mg/dL or HbA1c < 5.7%, and prediabetes group was defined as a FPG of 100-125 mg/dL or HbA1c of 5.7-6.4%. Diabetes group was defined as FPG ≥ 126 mg/dL or HbA1c ≥ 6.5% [2].

### Measurements

Socio-demographic characteristics including gender, age, education level, marital status, family CVD history, economic activity, and income level were analyzed. Family CVD history was defined as having a parent who was diagnosed with one or more of the following illnesses: hypertension, diabetes, ischemic heart disease, or stroke. Stress was classified based on a survey parameter asking the participants about their perceived stress, where those who perceived high stress marked “yes” and others marked “no.” We divided physical inactivity into PA and sedentary behavior for analysis. PAs were surveyed using the Global Physical Activity Questionnaire (GPAQ), and the PA in three domains (work, transport, and discretional) were used. As per the GPAQ analysis guide published by WHO, the measurements were converted to minutes and the amount of exercise was calculated as metabolic equivalent (MET). Total PA score was obtained by adding the scores for vigorous intensity activity (MET [8.0× min× d]), moderate intensity activity, and transport (MET [4.0× min× d]). The level of PA was classified into light intensity activity (< 3.0 METs), moderate intensity activity (3.0-6.0 METs), and vigorous intensity activity (≥ 6.0 METs) based on the MET results [23]. Sedentary behavior was assessed using the sedentary time surveyed in the GPAQ. The cut-off for sedentary time was set to 8 hours based on a previous study [12]. Unhealthy eating habits were analyzed based on breakfast frequency and the frequency of eating out. Eating out included delivered food, to-go, and meals from group meal services.

### Data analysis

The data were analyzed using SPSS version 21 (IBM Corp., Armonk, NY, USA). Normal and prediabetes groups and normal and diabetes groups were compared using univariable analysis including independent t-tests and chi-square tests. To identify the lifestyle-related predictors of prediabetes and diabetes, multivariable logistic regression was performed with the variables that were found to be significant in comparison with the normal group, considered as the independent variables. Statistical significance was set at p-value< 0.05.

### Ethics statement

This is a secondary data analysis, so ethical review and approval were not required. We submitted a request for raw data on the KNHANES website (http://knhanes.cdc.go.kr) and obtained the raw data, stripped of personal information upon approval.

## RESULTS

### Comparison of sample characteristics among the normal, prediabetes, and diabetes groups

When participants were classified based on the FPG and HbA1c levels, 77.4% were in the normal group, 21.2% in the prediabetes group, and 1.4% in the diabetes group. Women accounted for the majority at 60.4% and 56.1% in the normal and diabetes groups, respectively, while men accounted for the majority at 56.5% in the prediabetes group. Smoking rate increased in the order of normal, prediabetes, and diabetes groups, with 36.5% smokers in the diabetes group. In contrast, sedentary behavior decreased in the order of normal, prediabetes, and diabetes groups, with an average of 8.5 hours in the normal group. PA increased in the order of normal, prediabetes, and diabetes groups, with 43.3 MET (hr/wk) in the diabetes group (Table 1).

### Prevalence of prediabetes and diabetes according to sample characteristics

The prevalence of prediabetes and diabetes was 27.7% and 1.4% in men, respectively and 16.3% and 1.3%, in women, respectively. In terms of demographic and lifestyle-related characteristics, the prevalence of prediabetes and diabetes was higher in people in their 30s, married, and people with a family CVD history in both men and women (Table 2).

### Comparison of sample characteristics between the normal and prediabetes, and the normal and diabetes groups

We compared the characteristics of the normal and prediabetes groups, which were classified according to FPG and HbA1c levels. The mean age of the normal group and prediabetes group was 29.7 years and 32.9 years, respectively, in men (p< 0.001); and 30.3 years and 32.7 years, respectively, in women (p< 0.001), showing that the prediabetes group was significantly older in both men and women. The percentage of high school graduates was higher in the prediabetes group (40.8%) than in the normal group (35.3%) in women (p< 0.05). BMI was significantly higher in the prediabetes group (men, 25.8 kg/m^2^; women, 24.0 kg/m^2^) than in the normal group (men, 23.9 kg/m^2^; women, 21.7 kg/m^2^) in both men (p<0.001) and women (p<0.001) (Table 3). We compared the characteristics of the normal group and diabetes group. The mean age was significantly higher among the diabetes group in both men (34.2 years, p< 0.001) and women (34.4 years, p< 0.001). BMI was significantly higher in the diabetes group in both men (27.9 kg/m^2^, p= 0.001) and in women (28.2 kg/m^2^, p< 0.001) (Table 4).

### Predictors of prediabetes and diabetes compared to the normal group

We performed logistic regression analysis to identify the lifestyle-related predictors of prediabetes and diabetes in comparison with the normal group. In men, age, BMI, and PA were identified as significant predictors. The odds of developing prediabetes increased by 1.10 times with an increase in age by 1- year (p< 0.001) and by 1.17 times with 1 kg/m^2^ increase in BMI (p< 0.001). Moreover, participants who engaged in light intensity activity (< 3.0 METs) were 1.55 times more likely to develop prediabetes than those who engaged in moderate-vigorous intensity activity (≥ 3.0 METs) (p= 0.002). Compared to the normal group, the lifestyle-related predictors of diabetes were age and BMI. The odds of developing diabetes from a normal state increased by 1.22 times with an increase in age by one year (p= 0.004) and by 1.28 times with 1 kg/m^2^ increase in BMI (p< 0.001) (Table 5).

In women, age, education level, and BMI were identified as statistically significant lifestyle-related predictors of prediabetes. The odds of developing prediabetes increased by 1.08 times with an increase in age by one year (p< 0.001), decreased by 0.77 times among college graduates or higher compared to high school graduates (p= 0.045), and increased by 1.16 times with one kg/m^2^ increase in BMI (p< 0.001). Compared to the normal group, age and BMI were identified as significant predictors of diabetes. The odds of developing diabetes increased by 1.16 times with an increase in age by one year (p= 0.001) and by 1.43 times with one unit increase in BMI (p< 0.001) (Table 5).

## DISCUSSION

Of the total study population, 27.7% of men and 16.3% of women were in the prediabetes group and 1.4% of men and 1.3% of women were in the diabetes group, and we will discuss the major findings according to gender.

First, regression revealed that PA is the most potent lifestyle-related predictor of progression to prediabetes in men. In other words, people who engage in light intensity activity were 1.55 times more likely to progress to prediabetes compared to people who engage in moderate-vigorous intensity activity. This supports the results of a meta-analysis where increased moderate intensity activity lowered FPG and HbA1c levels [5]. Further, it is in line with the WHO recommendation of engaging in at least 30 minutes of moderate-intensity activity to prevent diabetes [1]. Particularly, in view of 27.7% of men participants having prediabetes with 77.0% in their 30s, promoting moderate intensity PA in young men in their 30s is crucial. However, univariate analysis showed that the percentage of people who engage in 8 hours or more of sedentary time a day was higher in the normal group among both men and women. This is contradictory to the previous results pertaining to sedentary behavior. According to previous studies, increased sedentary time elevates the risk of diabetes and CVD [7-9]. The inconsistency in the results may be attributable to the fact that we obtained sedentary time measured by a self-reported survey, which can hinder obtaining accurate duration. Although there are many ongoing studies on sedentary behavior, the standard for sedentary behavior is yet to be derived, as suggested by the American Heart Association [24], so additional studies on PA and sedentary behavior are needed to establish sufficient evidence. In the future, in-depth exploratory studies are needed to investigate the association between sedentary behavior and PAs and these studies should utilize an accelerometer to take objective measurements.

BMI was identified as a significant lifestyle-related predictor of prediabetes as well as diabetes in both men and women. In other words, the odds of progressing to prediabetes and diabetes increased by 1.17 times and 1.28 times, respectively, in men; and by 1.16 times and 1.43 times, respectively, in women, with increasing BMI. This is similar to a previous finding that the odds to progress to prediabetes and diabetes is 1.91 times and 1.93 times, respectively, with increasing BMI [13], yet our results seem to show lower ORs due to a narrower range of age. A study on young adults with an average age of 24 years found that the odds to progress to prediabetes and diabetes increased by 1.14 times [25], which is similar to our findings. As shown here, we confirmed that higher BMI is a harmful factor leading to prediabetes and diabetes in young men and women. However, obesity rate in adults is continuously rising with the highest obesity rate among the 30s, besides the consumption of fat, drinks, non-home-cooked foods, and convenience foods is consistently growing [11]. Moreover, with rising one-person households among young adults and high accessibility to places to eat out and convenience foods, obesity is projected to worsen in the coming years. Therefore, effective measures to manage obesity rate among young adults are essential to prevent progression to prediabetes and diabetes.

There were no significant differences in unhealthy eating habits between the normal group with prediabetes and diabetes groups in both men and women. In a previous study, poor diet increased BMI and waist circumference in the breakfast skipping-high eating out group [15], and breakfast skipping elevated the risk for type 2 diabetes [16], which differed from our findings. The reason for this inconsistency can be attributed to the limitations of the categories of responses to items, as we defined eating out twice or more a day as the high eating out group. This means that people in their 20s and 30s today commonly eat non-home-cooked foods twice or more a day, which includes delivery food, to-go, and group meal services, and thus the current definition for the high eating out group has low discriminatory power. Furthermore, according to a study that used the same classification for eating out [15], frequent eat-outs increase BMI and waist circumference, but this result did not contribute to diabetes. Moreover, the findings of this study seem to differ from our study, as they enrolled participants older than 20 years of age while we examined young adults. Therefore, a more detailed survey pertaining to eating out should be utilized to analyze the growing frequency of eating out in today’s society. Unhealthy eating habits should also be analyzed based on an array of factors related to diet, as the people today show increased fat, drink, and sodium intake with reduced fruit intake.

Low education level was identified as a predictor of progression to prediabetes in women. In other words, college graduates or higher were 0.77 times less likely to progress to prediabetes compared to high school graduates. This is similar to previous findings that socioeconomic status (SES), such as household income and education level, is closely associated with diabetes [26]. Hence, people with low SES, particularly less educated women, should be taken into consideration when devising policies for preventing and managing prediabetes and diabetes. Further studies should be conducted to examine how diabetes is associated with economic activity and household income as well as education level.

Regarding smoking and drinking, univariate analysis showed that there was a significant difference in smoking between the normal group and prediabetes group among men but not among women. Previous studies reported that smoking is strongly associated with diabetes [14,17,18], although in our study, smoking was not a predictor of prediabetes and diabetes. We speculate that this is because our study was a cross-sectional study, which cannot describe causal relationships. Drinking significantly differed between the normal and prediabetes groups among women alone, but there was no significant difference between the normal and diabetes groups. Previous studies found a close association between drinking and diabetes [25], but our results do not support this. This can be attributed to our study population that consisted of young adults and thus only a small number of people belonged in the diabetes group. Moreover, we compared drinking with a group that never drank in the past year, which limits direct comparison. Because excessive alcohol consumption is a risk factor of diabetes, and binge drinking rate is high among Korean young adults, further studies are needed to examine the association between alcohol consumption and prediabetes and diabetes in young adults.

The significance of this study is that it analyzed some of the key lifestyle-related factors, such as smoking, drinking, obesity, PA, and sedentary behavior among young adults, an age group in which change can be introduced with lifestyle modification. Furthermore, as we confirmed that PA and obesity are the most potent predictors of prediabetes and diabetes in young adults, which are modifiable, our findings present evidence highlighting the importance of lifestyle management. In addition, the use of KNHANES, a nationally representative data, renders our findings generalizable to the entire Korean population. However, we could not control for the new emerging lifestyle-related risk factors such as social participation and sleep duration, homological factors such as blood pressure, cholesterol, and triglycerides, and psychological factors such as depression in our analysis. Besides, we used a cross-sectional survey conducted at one-time point using a selfreported questionnaire, and unavailable missing data were excluded from the raw data, necessitating caution when generalizing the results as predictors of the entire Korean population.

This study underlines the importance of obesity management to prevent prediabetes and diabetes in young adults as well as the need for strategies tailored to less educated young women and less physically active men in their 30s to effectively lower obesity by promoting PAs. Lifestyle-related characteristics such as eating patterns, PAs, and sedentary behaviors in young adults should be investigated through in-depth exploratory studies involving interviews, and replication studies utilizing objective instruments should be performed.

## Figures and Tables

**Table 1.** Comparison of subject characteristics of three groups with fasting blood sugar and hemoglobin levels (n=4,190)

Variables		Normal	Prediabetes	Diabetes
Total		3,243 (77.4)	890 (21.2)	57 (1.4)
Gender	Men	1,285 (39.6)	503 (56.5)	25 (43.9)
	Women	1,958 (60.4)	387 (43.5)	32 (56.1)
Age (yr)		30.05±5.80	32.83±5.10	34.32±4.56
	20-29	1,457 (44.9)	205 (23.0)	8 (14.0)
	30-39	1,786 (55.1)	685 (77.0)	49 (86.0)
Education	High school	1,200 (39.3)	330 (40.5)	24 (50.0)
	≥College	1,855 (60.7)	485 (59.5)	24 (50.0)
Marital status	Married	1,599 (49.3)	584 (65.6)	41 (71.9)
CVD family history	Yes	1,556(48.8)	488 (55.5)	37 (67.3)
Current job	Yes	1,956 (63.9)	557 (70.7)	33 (68.8)
Monthly household income (104 Korean won)		456.59±282.95	435.88±258.53	375.99±213.06
	1st quartile	219 (6.8)	46 (5.2)	8 (14.0)
	2nd quartile	746 (23.1)	219 (24.7)	13 (22.8)
	3rd quartile	1,150 (35.6)	342 (38.5)	22 (38.6)
	4th quartile	1,118 (34.6)	281 (31.6)	14 (24.6)
BMI (kg/m^2^)		22.58±3.46	25.04±4.16	28.11±5.07
Perceived stress	Yes	193 (6.1)	59 (6.9)	5 (9.6)
Current smoker	Yes	687 (21.7)	288 (33.5)	19 (36.5)
Current drinker (times/yr)	≥1	2,739 (86.4)	737 (85.8)	42 (80.8)
Unhealthy eating habit				
Eating out (times/d)	≥2	2,465 (88.2)	635 (84.9)	46 (92.0)
Having breakfast (times/wk)	≥3	1,629 (58.3)	411 (54.9)	25 (50.0)
Sedentary behavior (hr/d)		8.52±3.58	8.10±3.64	7.39±3.06
	<8	1,205 (39.4)	366 (45.0)	21 (44.7)
	≥8	1,850 (60.6)	447 (55.0)	26 (55.3)
Physical activity, MET (hr/wk)		30.35±39.23	34.35±54.27	43.31±71.29
	Light	1,350 (55.9)	363 (59.8)	15 (46.9)
	Moderate to vigorous	1,065 (44.1)	244 (40.2)	17 (53.1)

Values are presented as number (%) or mean±standard deviation. CVD, cardiovascular disease; BMI, body mass index; MET, metabolic equivalent.

**Table 2.** Prevalence^1^ of prediabetes and diabetes according to lifestyle variables

Variables		Men (n=1,813)		Women (n=2,377)	
		Prediabetes	Diabetes	Prediabetes	Diabetes
Total		503 (27.7)	25 (1.4)	387 (16.3)	32 (1.3)
Age (yr)	20-29	111 (6.1)	3 (0.2)	94 (4.0)	5 (0.2)
	30-39	392 (21.6)	22 (1.2)	293 (12.3)	27 (1.1)
Education	High school	179 (10.8)	11 (0.7)	151 (6.7)	13 (0.6)
	≥College	266 (16.0)	9 (0.5)	219 (9.7)	15 (0.7)
Marital status	Married	303 (16.7)	15 (0.8)	281 (11.8)	26 (1.1)
	Unmarried	200 (11.0)	10 (0.6)	105 (4.4)	6 (0.3)
CVD family history	Yes	262 (14.8)	17 (1.0)	226 (9.6)	20 (0.9)
	No	236 (13.3)	7 (0.4)	156 (6.6)	11 (0.5)
Current job	Yes	388 (23.3)	18 (1.1)	189 (8.4)	15 (0.7)
	No	58 (3.5)	2 (0.1)	181 (8.0)	13 (0.6)
BMI (kg/m^2^)	<25	221 (12.2)	6 (0.3)	252 (10.6)	9 (0.4)
	≥25	278 (15.4)	19 (1.1)	135 (5.7)	23 (1.0)
Perceived stress	Yes	37 (2.1)	1 (0.1)	22 (0.9)	4 (0.2)
	No	444 (25.3)	21 (1.2)	356 (15.3)	26 (1.1)
Current smoker	Yes	253 (14.4)	14 (0.8)	35 (1.5)	5 (0.2)
	Ex- or never	228 (13.0)	8 (0.5)	343 (14.8)	25 (1.1)
Current drinker	≥1 time/yr	446 (25.4)	20 (1.1)	291 (12.5)	22 (0.9)
	Ex- or never	35 (2.0)	2 (0.1)	87 (3.7)	8 (0.3)
Unhealthy eating habit					
Eating out (times/d)	≥2	302 (20.7)	17 (1.2)	333 (15.6)	29 (1.4)
	<2	99 (6.8)	4 (0.3)	14 (0.7)	1 (0.0)
Having breakfast (times/wk)	≥3	206 (14.1)	9 (0.6)	205 (9.6)	16 (0.7)
	<3	195 (13.4)	12 (0.8)	142 (6.7)	13 (0.6)
Sedentary behavior (hr/d)	<8	192 (11.5)	10 (0.6)	174 (7.7)	11 (0.5)
	≥8	254 (15.3)	10 (0.6)	193 (8.6)	16 (0.7)
Physical activity, MET (hr/wk)	Light	183 (14.1)	4 (0.3)	180 (10.3)	11 (0.6)
	Moderate to vigorous	141 (10.9)	7 (0.5)	103 (5.9)	10 (0.6)

Values are presented as number (%). CVD, cardiovascular disease; BMI, body mass index; MET, metabolic equivalent.

1 Divided by the total number of men or women.

**Table 3.** Differences in subject characteristics and lifestyle variables between normal and prediabetic groups

Variables		Men (n=1,788)			Women (n=2,345)		
		Normal (n=1,285)	Prediabetes (n=503)	p-value^1^	Normal (n=1,958)	Prediabetes (n=387)	p-value^1^
Age (yr)		29.67±5.76	32.90±5.00	<0.001	30.30±5.82	32.75±5.24	<0.001
Education	High school	544 (45.4)	179 (40.2)	0.060	656 (35.3)	151 (40.8)	0.045
	≥College	654 (54.6)	266 (59.8)		1,201 (64.7)	219 (59.2)	
Marital status	Married	515 (40.1)	303 (60.2)	<0.001	1,084 (55.4)	281 (72.6)	<0.001
	Unmarried	770 (59.9)	200 (39.8)		874 (44.6)	105 (27.1)	
CVD family history	Yes	551 (43.9)	262 (52.6)	<0.001	1,005 (51.9)	226 (59.2)	0.009
	No	703 (56.1)	236 (47.4)		931 (48.1)	156 (40.8)	
Current job	Yes	899 (74.9)	388 (87.0)	<0.001	1,057 (56.9)	189 (51.1)	0.041
	No	302 (25.1)	58 (13.0)		802 (43.1)	181 (48.9)	
Monthly household income (10^4^ Korean won)		454.25±287.52	433.41±253.25	0.133	458.12±279.97	439.12±265.57	0.220
	BMI (kg/m^2^)	23.94±3.47	25.85±3.71	<0.001	21.69±3.144	24.00±4.48	<0.001
Perceived stress	Yes	62 (4.9)	37 (7.7)	0.027	131 (6.8)	22 (5.8)	0.472
	No	1,191 (95.1)	444 (92.3)		1,787 (93.2)	356 (94.2)	
Current smoker	Yes	554 (44.2)	253 (52.6)	0.002	133 (6.9)	35 (9.3)	0.113
	Ex- or never	699 (55.8)	228 (47.4)		1,784 (93.1)	343 (90.7)	
Current drinker	≥1 time/yr	1,171 (93.5)	446 (92.7)	0.586	1,568 (81.8)	291 (77.0)	0.031
	Ex- or never	82 (6.5)	35 (7.3)		350 (18.2)	87 (23.0)	
Unhealthy eating habit							
Eating out (times/d)	≥2	806 (77.7)	302 (75.3)	0.329	1,659 (94.3)	333 (96.0)	0.214
	<2	231 (22.3)	99 (24.7)		100 (5.7)	14 (4.0)	
Having breakfast (times/wk)	≥3	581 (56.0)	206 (51.4)	0.112	1,048 (59.6)	205(59.1)	0.862
	<3	456 (44.0)	195 (48.6)		711 (40.4)	142 (40.9)	
Sedentary behavior (hr/d)	<8	444 (37.1)	192 (43.0)	0.027	761 (41.0)	174 (47.4)	0.022
	≥8	753 (62.9)	254 (57.0)		1,097 (59.0)	193 (52.6)	
Physical activity, MET (hr/wk)	Light	446 (46.3)	183 (56.5)	0.002	904 (62.3)	180 (63.6)	0.669
	Moderate to vigorous	517 (53.7)	141 (43.5)		548 (37.7)	103 (36.4)	

Values are presented as number (%) or mean±standard deviation. CVD, cardiovascular disease; BMI, body mass index; MET, metabolic equivalent.

1 Calculated by t-test or chi-square test.

**Table 4.** Differences in subject characteristics and lifestyle variables between normal and diabetic groups

Variables		Men (n=1,310)			Women (n=1,990)		
		Normal (n=1,285)	Diabetes (n=25)	p-value^1^	Normal (n=1,958)	Diabetes (n=32)	p-value^1^
Age (yr)		29.67±5.76	34.24±4.22	<0.001	30.30±5.82	34.38±4.89	<0.001
Education	High school	544 (45.4)	11 (55.0)	0.393	656 (35.3)	13 (46.4)	0.223
	≥College	654 (54.6)	9 (45.0)		1,201 (64.7)	15 (53.6)	
Marital status	Married	515 (40.1)	15 (60.0)	0.044	1,084 (55.4)	26 (81.2)	0.003
	Unmarried	770 (59.9)	10 (40.0)		874 (44.6)	6 (18.8)	
CVD family history	Yes	551 (43.9)	17 (70.8)	0.009	1,005 (51.9)	20 (64.5)	0.163
	No	703 (56.1)	7 (29.2)		931 (48.1)	11 (35.5)	
Current job	Yes	899 (74.9)	18 (90.0)	0.189	1,057 (56.9)	15 (53.6)	0.727
	No	302 (25.1)	2 (10.0)		802 (43.1)	13 (46.4)	
Monthly household income (10^4^ Korean won)		454.25±287.52	458.12±279.97	0.157	458.12±279.97	378.82±214.68	0.111
BMI (kg/m^2^)		23.94±3.47	27.94±5.01	0.001	21.69±3.14	28.25±5.19	<0.001
Perceived stress	Yes	1,191 (95.1)	21 (95.5)	1.00	1,787 (93.2)	26 (86.7)	0.150
	No	62 (4.9)	1 (4.5)		131 (6.8)	4 (13.3)	
Current smoker	Yes	554 (44.2)	14 (63.6)	0.069	133 (6.9)	5 (16.7)	0.056
	Ex- or never	699 (55.8)	8 (36.4)		1,784 (93.1)	25 (83.3)	
Current drinker	≥1 time/yr	1,171 (93.5)	20 (90.9)	0.652	1,568 (81.8)	22 (73.3)	0.237
	Ex- or never	82 (6.5)	2 (9.1)		350 (18.2)	8 (26.7)	
Unhealthy eating habit							
Eating out (times/d)	≥2	231 (22.3)	4 (19.0)	1.00	100 (5.7)	1 (3.3)	0.404
	<2	806 (77.7)	17 (81.0)		1,659 (94.3)	29 (96.7)	
Having breakfast (times/wk)	≥3	581 (56.0)	9 (42.9)	0.230	1,048 (59.6)	16 (55.2)	0.632
	<3	456 (44.0)	12 (57.1)		711 (40.4)	13 (44.8)	
Sedentary behavior (hr/d)	<8	444 (37.1)	10 (50.0)	0.237	761 (41.0)	11 (40.7)	1.00
	≥8	753 (62.9)	10 (50.0)		1,097 (59.0)	16 (59.3)	
Physical activity, MET (hr/wk)	Light	446 (46.3)	4 (36.4)	0.560	904 (62.3)	11 (52.4)	0.371
	Moderate to vigorous	517 (53.7)	7 (63.6)		548 (37.7)	10 (47.6)	

Values are presented as number (%) or mean±standard deviation. CVD, cardiovascular disease; BMI, body mass index; MET, metabolic equivalent.

1 Calculated by t-test or chi-square test.

**Table 5.** Comparison of lifestyle predictors affecting prediabetes and diabetes group compared to normal group by gender

Variables		Men	p-value	Women	p-value
Normal vs. prediabetes^1^					
Age (yr)		1.10 (1.07, 1.14)	<0.001	1.08 (1.04, 1.11)	<0.001
Education	High school	-		1.00 (reference)	
	≥College	-		0.77 (0.60, 0.99)	0.045
Marital status	Unmarried	1.00 (reference)		1.00 (reference)	
	Married	0.99 (0.69, 1.43)	0.965	1.04 (0.73, 1.49)	0.831
CVD family history	No	1.00 (reference)		1.00 (reference)	
	Yes	1.13 (0.86, 1.49)	0.396	1.12 (0.88, 1.42)	0.376
Current job	Yes	1.00 (reference)		1.00 (reference)	
	No	1.02 (0.67, 1.55)	0.934	1.09 (0.85, 1.39)	0.508
BMI (kg/m^2^)		1.17 (1.11, 1.21)	<0.001	1.16 (1.12, 1.20)	<0.001
Perceived stress	No	1.00 (reference)		-	
	Yes	1.26 (0.71, 2.21)	0.429	-	
Current smoker	Ex- or never	1.00 (reference)		-	
	Yes	1.28 (0.97, 1.69)	0.085	-	
Drinking	Ex- or never	-		1.00 (reference)	
	Current drinker	-		0.82 (0.62, 1.09)	0.166
Sedentary behavior (hr/d)	<8	1.00 (reference)		1.00 (reference)	
	≥8	0.78 (0.58, 1.04	0.087	0.91 (0.72, 1.17)	0.472
Physical activity, MET (hr/wk)	Moderate to vigorous	1.00 (reference)		-	
	Light	1.55 (1.17, 2.05)	0.002	-	
Normal vs. diabetes^2^					
Age (yr)		1.22 (1.07, 1.41)	0.004	1.16 (1.06, 1.26)	0.001
Marital status	Unmarried	1.00 (reference)		1.00 (reference)	
	Married	1.44 (0.45, 4.56)	0.537	0.97 (0.30, 3.18)	0.962
CVD family history	No	1.00 (reference)		-	
	Yes	2.45 (0.98, 6.13)	0.056	-	
BMI (kg/m^2^)		1.28 (1.15, 1.42)	<0.001	1.43 (1.31, 1.55)	<0.001

Values are presented as adjusted odds ratio (95% confidence interval). BMI, body mass index; CVD, cardiovascular disease; MET, metabolic equivalent.

1 Men: adjusted for age, marital status, CVD family history, current job, BMI, stress, smoking, sedentary behavior, physical activity; Women: adjusted for age, education, marital status, CVD family history, current job, BMI, drinking, sedentary behavior.

2 Men: adjusted for age, marital status, CVD family history, BMI; Women: adjusted age, marital status, BMI.

## SUPPLEMENTARY MATERIALS

Korean version is available at http://www.e-epih.org/.
