# Supplementary material for: Lifestyle-related predictors affecting prediabetes and diabetes in 20-30-year-old young Korean adults
Source: Epidemiol Health. 2020 Mar 19;42:e2020014. doi: 10.4178/epih.e2020014 (PMC7285443; doi:10.4178/epih.e2020014)
Supplement: Supplementary file 1 [file epih-42-e2020014-suppl.docx]

**Lifestyle-related predictors affecting prediabetes and diabetes in 20-30-year-old young Korean adults**

**ABSTRACT**

**OBJECTIVES:** 이 연구는 2014-2016년도 국민건강영양조사 자료를 이용하여 만 20-39세 젊은 성인에서 성별에 따라 당뇨전단계와 당뇨를 예측하는 생활습관 관련 요인을 확인하고자 하였다.

**METHODS:** 국민건강영양조사자료의 이차분석을 통한 단면적 조사연구이다. 공복혈당 수치와 당화혈색소 수치를 바탕으로 정상군 (공복혈당 <100 ㎎/㎗ and/or 당화혈색소 <5.7%), 당뇨전단계군 (공복혈당 100~125 ㎎/㎗ and/or 당화혈색소 5.7%~6.4%), 당뇨군(공복혈당 ≥126 ㎎/㎗ and/or 당화혈색소 ≥6.5%)으로 구분하였으며, SPSS를 이용하여 통계분석을 하였다.

**RESULTS:** 총 4,190명의 대상자 중에서 남성 27.7%, 여성 16.3%가 당뇨전단계, 남성 1.4%, 여성1.3%가 당뇨단계로 확인되었다. 로지스틱 회귀분석 결과, 남성과 여성 모두에서 연령과 비만도는 당뇨전단계 및 당뇨단계의 예측요인으로 나타났으며, 낮은 신체활동은 남성에서, 낮은 교육수준은 여성에서 당뇨전단계의 예측인자로 확인되었다(*p*<.05).

**CONCLUSIONS:** 20-30대의 젊은 성인 남녀에서 연령과 비만도의 증가가 혈당을 올리는 예측인자임을 알 수 있었으며, 특히 30대 남성에서 신체활동을 증진을 통한 비만도를 낮추는 전략이 대사증후군을 예방하고 당뇨전단계로의 진행을 막기위해 절대적으로 필요함을 확인하였다.

KEY WORDS: Diabetes, Pre-diabetes, Lifestyle, Physical inactivity, Sedentary behavior, Young adult

**INTRODUCTION**

전 세계적으로 당뇨병의 유병률은 지속적으로 증가하였으며[1] 한국의 경우 연령이 증가함에 따라 남녀 모두 유병률이 증가하였다. 30세 이상 성인의 약 14.4%가 당뇨병이 있으며, 당뇨전단계의 유병률 또한 남성 31.0%, 여성 19.7%로 고위험상태라고 볼 수 있다. 아직까지 제 2형 당뇨병은 40대 이상에서 높은 유병률을 보이는 질환이나, 최근 당뇨전단계 유병률이 젊은 성인에서 증가하며[2] 전 세계적으로도 제 2형 당뇨병의 발생 연령이 낮아지고 있어[1] 더 이상 중년의 질환으로만 볼 수 없다. 특히, 경제활동이 가장 활발하지만 건강관리에 가장 취약한 30대는 당뇨병 환자 약 50% 정도만 당뇨병을 인지하고 있으며[3], 당뇨전단계의 유병률은 당뇨병에 비해 약 5배 이상 높은 수치를 보였다[2]. 또한, 건강검진 수진율도 다른 연령층에 비해 낮아 유병률 확인조차 어려운 상황이다[4]. 이처럼 젊은 성인은 당뇨병에 대한 인지와 관리가 매우 부족한 실정이라 일차 예방적 차원에서 특히 이들에 대한 관심이 필요하다. 또한, 공복혈당 뿐만 아니라 당뇨전단계 및 당뇨병의 진단에 유용성이 확인되어 2018년부터 당뇨병 진단 기준에 추가된 당화혈색소(HbA1c)를 포함하여 그 유병률과 관련 요인을 확인할 필요가 있다[2].

신체활동 부족은 당뇨병과 밀접한 관련이 있어 국제보건기구(World Health Organization, WHO)는 최소한 30분 동안 중등도 신체활동을 증진시켜야 한다고 권고하고 있다[1]. 체계적 문헌고찰에 따르면 중등도의 신체활동 증가는 공복혈당 및 당화혈색소를 감소시키는 효과가 있었다[5]. 최근 새로운 생활습관 위험요소로 좌식행동이 포함되었다[6]. 교통 및 업무 현장의 기술 발전으로 인하여 좌식행동 패턴은 널리 퍼져 있으며, 특히 고소득국가의 성인에서는 깨어 있는 많은 시간을 좌식행동으로 소비하고 있다[7]. 다양한 연구들에서 좌식시간이 늘어날수록 당뇨 및 심혈관질환의 위험이 높아지는 것으로 나타났다[8-9]. 하지만, 추후 젊은 성인기로 이행될 청소년의 신체활동 실천율은 감소하였고[10], 성인의 걷기 실천율, 유산소 및 근력운동 실천율도 감소하였다[11]. 또한, 한국은 긴 근무 시간과 초고속 인터넷을 사용하는 국가로, 매우 높은 좌식행동의 패턴을 보였다[12]. 따라서, 학업과 경제활동이 활발하고, 인터넷을 많이 사용하는 젊은 성인의 신체활동 부족은 추후 당뇨전단계 및 당뇨병 이환의 위험성을 높일 수 있으므로 조기에 예방 및 관리가 필요하다고 볼 수 있다.

비만을 분류하기 위한 가장 널리 사용되고 있는 체질량지수(Body Mass Index, BMI)가 증가할수록 당뇨전단계 및 당뇨의 위험이 증가하며[13], 지속적인 체중 감소는 당뇨전단계 및 제 2형 당뇨병의 진행을 지연시켰다[14], 특히 잦은 외식과 아침식사 결식은 당화혈색소 및 공복혈당을 상승시키며 제 2형 당뇨병과도 밀접한 연관성이 있다[15-16]. 최근, 성인 남성의 41.6%, 여성의 25.6%가 비만이며, 특히 30대의 비만율은 46.7%로 다른 연령군에 비해 가장 높았다. 또한 지방, 음료, 나트륨의 과잉섭취, 외식 및 편의 식품의 증가 등의 건강하지 않은 식이습관을 보였다[11]. 따라서 젊은 성인의 불건강한 식이습관과 과체중이 혈당에 어떠한 영향을 미치는지 확인할 필요가 있다.

흡연은 제 2형 당뇨병과 매우 밀접한 관련이 있는 것으로 나타났다[14, 17-18]. 능동적이고 수동적인 흡연은 제 2형 당뇨병의 위험을 증가시켰고[17], 제 2형 당뇨병이 있는 흡연자는 비흡연자에 비해 미세혈관 합병증 및 혈당 조절의 위험이 높음을 확인하였다[14]. 체계적 문헌고찰에 따르면 흡연자가 비흡연자에 비해 제 2형 당뇨병의 위험이 38% 더 높았으며, 과거 흡연자는 비흡연자에 비해 위험성이 19% 더 높음을 확인하였다[18]. 알코올과 당뇨병은 U자형의 관계를 가지고 있어 과도한 알코올 섭취(high alcohol consumption)는 당뇨병의 위험 요인으로 보고되었다[19]. 독특한 음주문화가 있는 한국은 남성이 여성에 비해 월간 폭음률이 약 2배 높으며, 20-30대 남성은 50% 이상의 월간 폭음률을, 20대 여성은 약 46%의 월간 폭음률을 보였다[11]. 과도한 알코올 섭취는 젊은 성인에게 당뇨병 이환의 위험성을 높일 수 있으므로 조기에 음주습관을 관리하는 것이 매우 중요하다고 볼 수 있다.

국내 젊은 성인을 대상으로 한 선행연구는 생활습관 요인에 중점을 두기보다는 생활습관과 지식과의 연관성[20], 당뇨자가관리의 관련요인[21], 당뇨병 임상적 특성[22]에 대한 연구로, 실제적으로 제 2형 당뇨병의 개선을 위해 관리를 할 수 있는 생활습관 요인을 확인한 선행연구는 매우 부족하였다. 따라서, 본 연구의 목적은 젊은 성인의 공복혈당 및 당화혈색소 정도에 따라 정상, 당뇨전단계 및 당뇨군으로 분류하고, 정상군과 비교하여 당뇨전단계와 당뇨군을 예측하는 생활습관 관련 요인을 확인하고자 한다. 이를 통해 당뇨병 이환의 일차적 예방을 위해 중년기 이전의 젊은 성인에서 생활습관 관리의 중요성에 대한 근거를 마련하고, 젊은 성인의 건강한 생활 유지에 기여하고자 한다.

**MATERIALS AND METHODS**

**Research design**

본 연구는 국민건강영양조사(Korea National Health and Nutrition Examination Survey, KNHANES) 자료를 이용한 이차분석 연구로, 20-30대의 젊은 성인의 당뇨전단계 및 당뇨군에 영향을 미치는 생활습관 요인을 확인하는 단면적 조사연구이다.

**Study population**

본 연구대상자는 2014-2016년도의 국민건강영양조사 원시자료를 활용하였다. 원시자료 23,080명의 대상자 중 연구에 필요한 만 20-39세로 연령을 제한하여 5,612명을 1차 선정하였다. 현병력은 의사의 진단을 받았거나 관련 약물을 복용하고 있는 대상자로 뇌졸중, 협심증, 심근경색, 당뇨의 현병력이 있는 대상자 513명을 제외하여 5,099명을 2차 선정하였으며, 공복혈당 수치 결측자 843명, HbA1c 수치 결측자 10명, 임신자 56명을 제외하여 총 4,190명을 연구 대상자로 선정하였다(Figure 1).

또한, 공복혈당 수치와 당화혈색소 수치를 바탕으로 정상군, 당뇨전단계군, 당뇨군으로 구분하였다. 공복혈당 수치 100 ㎎/㎗ 미만 또는 당화혈색소 수치 5.7% 미만인 경우를 정상군, 공복혈당 수치 100-125 ㎎/㎗ 또는 당화혈색소 수치 5.7-6.4% 인 경우를 당뇨전단계군, 공복혈당 수치 126 ㎎/㎗ 이상 또는 당화혈색소 수치 6.5% 이상인 경우를 당뇨군으로 정의하였다[2].

**Ethics statement**

본 연구의 자료는 2차 자료를 활용한 연구이므로 윤리적인 승인이 필요하지 않고, 국민건강영양조사 웹 사이트를 통해 신청을 하여 승인받은 후 대상자의 개인 정보가 삭제된 원시자료를 받았다.

**Measurements**

대상자의 인구사회학적 특성으로 성별, 연령, 교육수준, 결혼상태, 심혈관질환 가족력, 경제활동, 소득 수준을 포함하여 분석하였다. 심혈관질환 가족력은 부모 중 한명이라도 고혈압이나 당뇨와 같은 선행질환, 허혈성심질환, 뇌졸중 중 한가지 이상 진단을 받은 적 있는 경우 가족력이 있는 것으로 하였다. 스트레스는 평소 스트레스를 인지하는 정도의 문항에서 대단히 많이 느끼는 경우는 “예”, 그 외의 경우는 “아니오”로 구분하였다. 신체활동 부족은 신체활동과 좌식행동으로 나누어 분석하였다. 신체활동은 국제신체활동 설문지(Global Physical Activity Questionnaire, GPAQ)로 조사하였으며, 일에 대한 신체활동, 장소이동 신체활동, 여가 신체활동의 결과값을 활용하였다. 세계보건기구의 GPAQ analysis guide에 따라 측정결과를 분(minutes)으로 환산한 후 운동의 양을 신진대사 해당치(metabolic equivalent, MET)로 계산하였다. 총 신체활동량 점수 계산은 고강도활동 (Vigorous Intensity Activity) MET(8.0 x min x day), 중강도활동(Moderate Intensity Activity) 및 장소이동(Transport) MET(4.0 x min x day)의 점수를 모두 합산하였다. MET의 결과에 따라 신체활동수준을 저강도 신체활동(Light intensity activity(<3.0 METs)), 중등도 신체활동(Moderate Intensity Activity(3.0 to <6.0 METs)), 고강도 신체활동(Vigorous Intensity Activity(≥6.0 METs))으로 분류하였다[23]. 좌식행동은 국제신체활동 설문지(Global Physical Activity Questionnaire, GPAQ)로 조사한 좌식시간의 결과값을 활용하였다. 선행연구의 결과를 바탕으로[12] 좌식시간의 기준점을 8시간으로 하였다. 불건강 식습관을 확인하기 위해 아침식사 여부 및 외식의 횟수를 활용하여 구분하였다. 외식은 배달음식, 포장음식, 급식 등을 포함한다.

**Data analysis**

본 연구의 자료분석은 SPSS program version 21을 이용하였다. 공복혈당과 당화혈색소를 바탕으로 분류한 3개 그룹에서 정상군과 당뇨전단계군, 정상군과 당뇨군 각각의 특성을 비교하기 위해 독립표본 T 검정(t-test), 카이제곱 검정(chi-square test) 등 단변량 분석(univariable analysis)을 실시하였다. 당뇨전단계와 당뇨단계에 영향을 미치는 생활습관 요인을 확인하기 위해 각각 단변량분석에서 정상군과의 비교를 통해 유의하게 나타난 변수를 독립변수로 투입하여 다중 로지스틱 회귀분석(Multivariable logistic regression)을 실시하였다. 통계적 유의성은 *p*<.05으로 정하였다.

**RESULTS**

1. 정상, 당뇨전단계, 당뇨군의 일반적 및 건강관련 특성 비교

공복혈당 수치와 당화혈색소 수치에 따라 분류하였을 때, 본 연구의 대상자는 정상 77.4%, 당뇨전단계 21.2%, 당뇨 1.4%였다. 정상, 당뇨군은 여성이 각각 60.4%, 56.1%로 높은 비율을, 당뇨전단계는 남성이 56.5%로 높은 비율을 보였다. 흡연은 당뇨군이 36.5%로 당뇨군으로 갈수록 비율이 높았다. 좌식행동은 정상군이 8.5시간으로 당뇨군으로 갈수록 줄어들었으며, 신체활동량은 당뇨군이 43.3 MET (hr/wk) 로 당뇨군으로 갈수록 늘어났다(Table 1).

1. 인구사회학적 및 생활습관 관련 특성에 따른 당뇨전단계와 당뇨의 유병률

남성에서 당뇨전단계 유병률 27.7%, 당뇨 유병률 1.4%이며, 여성에서 당뇨전단계 유병률 16.3%, 당뇨 유병률 1.3%였다. 인구사회학적 및 생활습관 관련 특성에 따라 살펴보면, 남녀 모두 30대인 경우, 기혼자인 경우, 심혈관 가족력이 있는 경우 당뇨전단계 및 당뇨 유병률이 높았다(Table 2).

1. 정상군과 당뇨전단계군, 정상군과 당뇨군의 인구사회학적 및 생활습관 관련 특성 비교

공복혈당 수치와 당화혈색소 수치에 따라 분류된 정상군과 당뇨 전단계군의 특성을 비교한 결과, 연령에서 남성의 정상군은 29.7세, 당뇨전단계군은 32.9세 (*p*<.001), 여성의 정상군은 30.3세, 당뇨전단계군은 32.8세로 (*p*<.001), 남녀 모두 당뇨전단계군이 정상군보다 연령이 유의하게 높았다. 여성의 학력에서 고졸자는 당뇨전단계군 40.8%로 정상군의 35.3%보다 더 높게 나타났다 ( *p*<.001). BMI에서 남성의 정상군은 23.9 ㎏/㎡, 당뇨전단계군은 25.9 ㎏/㎡ (*p*<.001), 여성의 정상군은 21.7 ㎏/㎡, 당뇨전단계군은 24.0 ㎏/㎡로 (*p*<.001), 남녀 모두 당뇨전단계군이 정상군보다 BMI가 유의하게 높았다(Table 3). 정상군과 당뇨군의 특성을 비교한 결과, 연령에서 남성 당뇨군은 34.2세 (*p*<.001), 여성 당뇨군은 34.4세로 (*p*<.001), 남녀 모두 당뇨군이 정상군보다 연령이 유의하게 높았다. BMI에서 남성 당뇨군은 27.9 ㎏/㎡ (*p*=0.001), 여성 당뇨군은 28.3 ㎏/㎡로 (*p*<.001), 남녀 모두 당뇨군이 정상군보다 BMI가 유의하게 높았다(Table 4).

1. 당뇨전단계과 당뇨의 예측요인 (정상군과의 비교)

남성에서 정상군과 비교하여 당뇨전단계 및 당뇨에 영향을 미치는 생활습관 관련 요인을 확인하기 위해 로지스틱 회귀분석 한 결과, 연령, BMI, 신체활동이 통계적으로 유의한 요인으로 확인되었다. 연령이 1세 증가할수록 정상군에서 당뇨전단계로 이환 될 확률이 1.10배 증가하였고 (*p*<.001), BMI가 1 ㎏/㎡ 증가할수록 1.17배 증가하였으며 (*p*<.001), 저강도 신체활동(<3.0 METs)을 하는 대상자는 중-고강도 신체활동(≥3.0 METs)을 하는 대상자에 비해 1.55배 높았다 (*p*=.002). 정상군과 비교하여 당뇨에 영향을 미치는 생활습관 관련 요인은 연령, BMI가 유의한 요인으로 확인되었다. 연령이 1세 증가할수록 정상군에서 당뇨군으로 이환 될 확률이 1.22배 증가하였고 (*p*=.004), BMI가 1 ㎏/㎡ 증가할수록 1.28배 증가하였다 (*p*<.001) (Table 5).

여성에서 정상군과 비교하여 당뇨전단계 및 당뇨에 영향을 미치는 생활습관 관련 요인을 확인하기 위해 로지스틱 회귀분석 한 결과, 연령, 교육수준, BMI가 통계적으로 유의한 요인으로 확인되었다. 연령이 1세 증가할수록 정상군에서 당뇨전단계로 이환 될 확률이 1.08배 증가하였고 (*p*<.001), 대졸 이상 대상자는 고졸 대상자에 비해 0.77배 감소하였고 (*p*=.045), BMI가 1 ㎏/㎡ 증가할수록 1.16배 증가하였다 (*p*<.001). 정상군과 비교하여 당뇨에 영향을 미치는 생활습관 관련 요인은 연령, BMI가 유의한 요인으로 확인되었다. 연령이 1세 증가할수록 정상군에서 당뇨군으로 이환 될 확률이 1.16배 증가하였고 (*p*=.001), BMI가 1단위 증가할수록 1.43배 증가하였다 (*p*<.001) (Table 5).

**DISCUSSION**

당뇨전단계군은는 남성 27.7%, 여성 16.3%, 당뇨군에는 남성 1.4%, 여성 1.3%가 포함되는 것으로 나타났으며, 성별에 따른 연구의 주요결과를 중심으로 논의하고자 한다.

먼저, 정상군과의 비교를 통한 당뇨전단계군 예측 요인에 대한 회귀분석 결과, 남성에서 당뇨전단계군으로 진행을 예측하는 가장 큰 생활습관 관련 요인으로 신체활동인 것으로 확인되었다. 즉, 저강도 신체활동을 하는 대상자는 중-고강도 신체활동을 하는 대상자에 비해 당뇨전단계로 이환될 확률이 1.55배 높았다. 이는 중등도 신체활동의 증가가 공복혈당 및 당화혈색소의 수치를 감소시킨다는 메타 분석 결과[5]를 지지한다. 또한 WHO가 최소 30분 동안의 중등도 신체활동을 증진시켜야 당뇨병 예방을 할 수 있다고 권고한 것과 맥락을 같이한다[1]. 특히 본 연구 남성 대상자의 27.7%가 당뇨전단계이고 이중 약 77%가 30대이므로 30대 젊은 남성을 대상으로 중등도 이상의 신체활동을 증진시키는 것이 절대적으로 필요하다고 본다. 하지만, 하루 좌식행동시간의 경우에는 단변량 분석 결과 남녀 모두 정상군에서 8시간 이상의 좌식행동 시간이 당뇨전단계군보다 더 길었다. 이는 좌식행동의 선행연구들과 상반된 결과이다. 선행연구 결과에 따르면 좌식행동 시간의 증가는 당뇨 및 심혈관질환의 위험을 증가시킨다[7-9]. 이러한 차이는 본 연구의 자료 수집이 자기보고식 설문지를 통해 좌식행동 시간을 확인하였기 때문에 정확한 시간을 측정하기 어려워 선행연구와 상반된 결과를 초래한 것으로 판단된다. 현재 많은 좌식행동 연구들이 진행되고 있으나 American Heart Association에서 제시하 듯[24] 아직 좌식행동의 기준이 되는 표준이 도출되지 않았기 때문에 더 많은 신체활동 및 좌식행동 연구를 통해 충분한 근거가 필요하다고 볼 수 있다. 추후 좌식행동과 신체활동의 관계에 대한 심층적 탐색 연구가 필요할 것이며, 객관적인 측정을 위해 가속도계를 활용한 연구가 진행되어야 할 것이다.

BMI는 남녀 모두에서 당뇨전단계뿐만 아니라 당뇨의 이환율을 높이는 유의한 생활습관 관련 요인으로 규명되었다. 즉, BMI가 증가할수록 당뇨전단계, 당뇨로 이환될 확률이 남성에서 1.17배, 1.28배, 여성에서 1.16배, 1.43배임을 확인하였다. 이는 BMI가 증가할수록 당뇨전단계, 당뇨로 이환될 확률이 1.91, 1.93배 증가한다는 선행연구결과[13]와 유사하나 연령의 범위가 넓지 않았기 때문에 교차비가 더 낮은 것으로 판단된다. 평균 24세의 성인초기 연구에서 당뇨전단계 및 당뇨로 이환될 확률이 1.14배 증가하여[25] 본 연구결과와 유사함을 확인하였다. 이처럼 BMI는 젊은 남성과 여성 모두에서 당뇨전단계 및 당뇨에 해로운 영향요인임을 확인하였다. 그러나, 성인의 비만율은 지속적으로 높아지고 있고, 특히 30대의 비만율이 가장 높으며, 지방 및 음료 섭취량, 외식 및 편의 식품의 섭취는 꾸준히 늘어나고 있다[11]. 또한, 젊은 연령대의 1인 가구의 증가와 외식 및 편의 식품의 접근성이 매우 편리하여 비만은 더 악화될 것으로 판단된다. 따라서, 추후 당뇨전단계 및 당뇨로 이환되지 않도록 젊은 연령층의 비만율을 감소시키기 위한 효과적인 관리방안이 반드시 필요하다고 본다.

하지만, 불량한 식습관의 변수는 정상과 당뇨전단계, 당뇨에서 남녀 모두 유의한 차이가 없었다. 선행연구에 따르면 아침 결식-고 외식군의 불량한 식습관이 BMI 및 허리둘레의 증가를 초래하고[15] 아침 결식이 제 2형 당뇨 발병 위험을 증가시켰으나[16], 본 연구결과와 달랐다. 그 이유는 문항을 구성하고 있는 범주의 한계로 고외식군에 대한 정의가 하루 2회 이상으로 제한된 것으로 꼽을 수 있다. 즉, 현 시대의 20-30대는 하루 2회 이상 배달음식, 포장음식, 급식 등의 외식을 하는 것이 보편화되어 있어 고외식군으로 범주화하기에 변별력이 떨어진다고 볼 수 있다. 또한, 동일하게 외식을 범주화한 연구에 따르면[15] 잦은 외식은 BMI, 허리둘레 증가를 초래하나, 이 결과가 당뇨에 영향을 미쳤다고 볼 수 없으며, 연령 또한 20대 이상을 대상자로 선정하였기 때문에 젊은 성인을 대상으로 한 본 연구결과와 다른 결과가 도출되었다고 판단된다. 따라서, 외식의 빈도가 증가하고 있는 현 시대에 더 세분화된 외식 문항으로 분석해 볼 필요성이 있으며, 지방, 음료, 나트륨의 섭취는 증가하고, 과일 섭취는 감소하고 있기 때문에 식습관과 관련된 다양한 변수를 활용하여 불건강한 식습관을 다시 분석할 필요가 있다.

낮은 교육수준은 여성에서 당뇨전단계군으로 진행을 예측하는 요인으로 확인되었다. 즉, 대졸 이상 대상자는 고졸 대상자에 비해 당뇨전단계로 이환될 확률이 0.77배 낮았다. 이는 가구소득, 교육 수준 등의 사회 경제적 지위(socio-economic status, SES)는 당뇨병과 밀접한 연관이 있다는 선행연구 결과[26]와 유사한 결과이다. 따라서, 당뇨전단계 및 당뇨병 예방 및 관리를 위해 사회 경제적 지위가 낮은 대상자, 특히 교육수준이 낮은 여성을 고려하여 정책을 수립해야 하며, 추후 세분화된 교육수준뿐만 아니라 경제활동, 가구소득과 당뇨병의 연관성을 확인하는 후속연구가 진행될 필요가 있다.

흡연과 음주의 경우 단변량 분석 결과를 살펴보면, 정상군과 당뇨전단계군 비교에서 남성은 흡연에 유의한 차이가 있었으나, 여성은 유의한 차이가 없었다. 선행 연구에서 흡연은 당뇨병과 매우 밀접한 관련이 있는 것으로 나타났으나[14, 17-18] 본 연구결과는 흡연이 당뇨전단계 및 당뇨의 이환율을 높이는 요인은 아니었다. 이는 본 연구가 단면연구로 흡연과 당뇨의 인과관계를 설명할 수 없기 때문으로 판단된다. 음주는 정상군과 당뇨전단계군의 비교에서 여성에서만 유의한 차이가 있었으나 정상군과 당뇨군의 비교에서 유의한 차이가 없었다. 선행 연구에서 음주는 당뇨병과 밀접한 관련성이 있었으나[25] 본 연구 결과는 이를 지지하지 못하였다. 이는 젊은 성인을 대상자로 하여 당뇨군에 해당되는 남녀의 수가 매우 적었으며, 본 연구에서는 음주를 1년 내에 전혀 하지 않는 군과의 비교를 한 것이기 때문에 직접 비교에는 제한이 있을 것으로 생각된다. 과도한 알코올 섭취는 당뇨병의 위험요인이며 한국 젊은 남녀의 폭음률이 높기 때문에 추후 후속연구를 통해 젊은 성인의 알코올 섭취와 당뇨전단계 및 당뇨병의 관계를 확인하는 연구가 필요하다.

본 연구는 생활습관 개선을 통해 충분히 변화의 가능성이 있는 젊은 성인을 대상으로 생활습관 핵심 구성 요소인 흡연, 음주, 비만, 신체활동뿐만 아니라 좌식행동을 함께 고려하여 분석하였다는데 의의가 있다. 또한, 젊은 성인의 당뇨전단계 및 당뇨에 영향을 미치는 가장 큰 영향 요인은 개선이 가능한 신체활동과 비만임을 확인하였기 때문에 추후 생활습관 관리가 매우 중요하다는 근거를 제공하였다는데 의의가 있다. 우리나라의 대표성을 가지고 있는 표본을 대상으로 실시한 국민건강영양조사 자료를 이용하여 분석하였기 때문에 연구결과를 전 국민에게 일반화할 수 있다는 장점이 있다. 하지만, 최근 생활습관의 위험요인으로 새롭게 부각되고 있는 사회적 참여도 및 수면시간, 혈압, 콜레스테롤, 중성지방 등의 혈액학적 요인 및 우울 등의 심리적 요인을 통제하지 못하고 분석하였다는 점과 일 시점에서의 자가보고식 설문에 의한 단면적 조사연구 자료이므로 인과관계를 설명할 수 없고, 원시자료에서 가용할 수 없는 결측 데이터(unavailable missing data)를 제외하였기 때문에 연구결과로 규명된 영향요인을 전 국민으로 일반화하는데 신중을 기하여야 할 것이다.

본 연구를 통해 젊은 성인 남녀의 당뇨전단계 및 당뇨의 이환을 예방하기 위해서는 비만 관리가 필수적이며, 특히 교육수준이 낮은 젊은 여성과 신체활동이 낮은 30대의 남성에서 신체활동 증진을 통해 효율적으로 비만도를 낮추는 전략 마련이 필요함을 확인하였다. 젊은 성인에서 식습관과 신체활동 및 좌식행동 등의 생활습관 관련 특성에 대해 인터뷰를 통한 심층 탐색연구와 그 유형을 확인할 필요가 있으며, 객관적 측정도구를 사용한 인구집단에서의 반복연구를 제언한다.

**CONFLICT OF INTEREST**

The authors have no conflicts of interest to declare for this study.

**REFERENCE**

1. World Health Organization. Diabetes fact sheet; 2019 [cited 2019 Aug 12] Available from https://www.who.int/news-room/fact-sheets/detail/diabetes.
2. Korean Diabetes Association. Diabetes fact sheet in Korea; 2018 [cited 2019 Aug 12] Available from http://www.diabetes.or.kr/pro/news/admin.php?category=A&code=admin&number=1546&mode=view.
3. Korea Centers for Disease Control and Prevention. Redcircle campaign; 2016 [cited 2019 Sep 2] Available from http://www.cdc.go.kr/board.es?mid=a20501000000&bid=0015&act=view&list_no=70792.
4. Kim CG, Lee SH, Cha SK. Influencing Factors on Cardio-cerebrovascular Disease Risk Factors in Young Men: Focusing on Obesity Indices. Journal of Korean Nursing Science 2017;19:1-10.
5. Boniol M, Dragomir M, Autier P, Boyle P. Physical activity and change in fasting glucose and HbA1c: a quantitative meta-analysis of randomized trials. Acta Diabetol 2017;54:983-991.
6. Krokstad S, Ding D, Grunseit AC, Sund ER, Holmen TL, Rangul V, et al. Multiple lifestyle behaviours and mortality, findings from a large population-based Norwegian cohort study - The HUNT Study. BMC Public Health 2017;17:58.
7. Ekelund U, Steene-Johannessen J, Brown WJ, Fagerland MW, Owen N, Powell KE, et al. Does physical activity attenuate, or even eliminate, the detrimental association of sitting time with mortality? A harmonised meta-analysis of data from more than 1 million men and women. Lancet 2016;388:1302-1310.
8. Patterson R, McNamara E, Tainio M, de Sá TH, Smith AD, Sharp SJ, et al. Sedentary behaviour and risk of all-cause, cardiovascular and cancer mortality, and incident type 2 diabetes: a systematic review and dose response meta-analysis. Eur J Epidemiol 2018;33:811-829.
9. Lim MS, Park B, Kong IG, Sim S, Kim SY, Kim JH, et al. Leisure sedentary time is differentially associated with hypertension, diabetes mellitus, and hyperlipidemia depending on occupation. BMC Public Health 2017;17:278.
10. Korea Centers for Disease Control and Prevention. Major results of Korea Youth Risk Behavior web-based Survey from 2018 (Korean, author’s translation) ; 2018 [cited 2019 Sep 2] Available from http://www.cdc.go.kr/board.es?mid=a20501000000&bid=0015&act=view&list_no=141752
11. Korea Centers for Disease Control and Prevention. Major results of Korea National Health and Nutrition Examination Survey from 2017 (Korean, author’s translation) ; 2018 [cited 2019 Sep 2] Available from http://www.cdc.go.kr/board.es?mid=a20501000000&bid=0015&act=view&list_no=141752
12. Nam JY, Kim J, Cho KH, Choi J, Shin J, Park EC. The impact of sitting time and physical activity on major depressive disorder in South Korean adults: a cross-sectional study. BMC Psychiatry 2017;17:274.
13. Liu L, Zhou C, Du H, Zhang K, Huang D, Wu J; Anshan Worker Health Survey Group. The prevalences of impaired fasting glucose and diabetes mellitus in working age men of North China: Anshan Worker Health Survey. Sci Rep 2014;4:4835.
14. American Diabetes Association. Lifestyle Management: Standards of Medical Care in Diabetes-2019. Diabetes Care 2019;42:S46-S60.
15. Lee JW. Effects of Frequent Eating-out and Breakfast Skipping on Body Mass Index and Nutrients Intake of Working Male Adults: Analysis of 2001 Korea National Health and Nutrition Survey Data. Korean journal of community nutrition 2009;14:789-797.
16. St-Onge MP, Ard J, Baskin ML, Chiuve SE, Johnson HM, Kris-Etherton P, et al. American Heart Association Obesity Committee of the Council on Lifestyle and Cardiometabolic Health; Council on Cardiovascular Disease in the Young; Council on Clinical Cardiology; and Stroke Council. Meal Timing and Frequency: Implications for Cardiovascular Disease Prevention: A Scientific Statement From the American Heart Association. Circulation 2017;135:e96-e121.
17. Pan A, Wang Y, Talaei M, Hu FB, Wu T. Relation of active, passive, and quitting smoking with incident type 2 diabetes: a systematic review and meta-analysis. Lancet Diabetes Endocrinol 2015;3:958-967.
18. Akter S, Goto A, Mizoue T. Smoking and the risk of type 2 diabetes in Japan: A systematic review and meta-analysis. J Epidemiol. 2017;27:553-561.
19. Hirst JA, Aronson JK, Feakins BG, Ma C, Farmer AJ, Stevens RJ. Short- and medium-term effects of light to moderate alcohol intake on glycaemic control in diabetes mellitus: a systematic review and meta-analysis of randomized trials. Diabet Med 2017;34:604-611.
20. Soh SY, Ma JE, Koh JY, Kim EH, Park HH, Seo YJ, et al. The Early adulthood’s life styles and knowledge about diabetic. Nurs Sci 2009;43:75-86.
21. Jung KM, Lee SJ, Park ES, Park YJ, Kim SG, Choi DS. Self-care and related factors in young and middle adulthood patients with type 2 diabetes. Journal of Korean Diabetes 2015;16:65-77.
22. Park MN, Kang YI, Chon S, Oh SJ, Woo JT, Kim SW, et al. The clinical characteristics of young onset diabetes according to etiology based classification. Diabetes and Metabolism Journal 2006;30:190-197.
23. Gibbs BB, Hergenroeder AL, Katzmarzyk PT, Lee IM, Jakicic JM. Definition, measurement, and health risks associated with sedentary behavior. Med Sci Sports Exerc 2015;47:1295-1300.
24. Young DR, Hivert MF, Alhassan S, Camhi SM, Ferguson JF, Katzmarzyk PT, et al. Physical Activity Committee of the Council on Lifestyle and Cardiometabolic Health; Council on Clinical Cardiology; Council on Epidemiology and Prevention; Council on Functional Genomics and Translational Biology; and Stroke Council Sedentary Behavior and Cardiovascular Morbidity and Mortality: A Science Advisory From the American Heart Association. Circulation 2016;134:e262-279.
25. Morrison JA, Glueck CJ, Umar M, Daniels S, Dolan LM, Wang P. Hyperinsulinemia and metabolic syndrome at mean age of 10 years in black and white schoolgirls and development of impaired fasting glucose and type 2 diabetes mellitus by mean age of 24 years. Metabolism 2011;60:24-31.
26. Kim YJ, Jeon JY, Han SJ, Kim HJ, Lee KW, Kim DJ. Effect of socio-economic status on the prevalence of diabetes. Yonsei Med J 2015;56:641-647.

Table 1. Comparison of subject characteristics of three groups with fasting blood sugar and HbA1c levels (n=4,190)

| Characteristics | Category | Normal | Pre-diabetes | Diabetes |
| --- | --- | --- | --- | --- |
|  |  | n=3,243 (77.4) | n=890 (21.2) | n=57 (1.4) |
|  |  | n (%) or M±SD | n (%) or M±SD | n (%) or M±SD |
| Gender | Male | 1285 (39.6) | 503 (56.5) | 25 (43.9) |
|  | Female | 1958 (60.4) | 387 (43.5) | 32 (56.1) |
| Age (years) |  | 30.05±5.80 | 32.83±5.10 | 34.32±4.56 |
|  | 20-29 | 1457 (44.9) | 205 (23.0) | 8 (14.0) |
|  | 30-39 | 1786 (55.1) | 685 (77.0) | 49 (86.0) |
| Education | High school | 1200 (39.3) | 330 (40.5) | 24 (50.0) |
|  | ≥College | 1855 (60.7) | 485 (59.5) | 24 (50.0) |
| Marital status | Married | 1599 (49.3) | 584 (65.6) | 41 (71.9) |
| CVD Family history | Yes | 1556(48.8) | 488 (55.5) | 37 (67.3) |
| Current job | Yes | 1956 (63.9) | 557 (70.7) | 33 (68.8) |
| Monthly household income (10,000 Won) |  | 456.59±282.95 | 435.88±258.53 | 375.99±213.06 |
|  | 1st quartile | 219 (6.8) | 46 (5.2) | 8 (14.0) |
|  | 2nd quartile | 746 (23.1) | 219 (24.7) | 13 (22.8) |
|  | 3rd quartile | 1150 (35.6) | 342 (38.5) | 22 (38.6) |
|  | 4th quartile | 1118 (34.6) | 281 (31.6) | 14 (24.6) |
| BMI (㎏/㎡) |  | 22.58±3.46 | 25.04±4.16 | 28.11±5.07 |
| Perceived stress | Yes | 193 (6.1) | 59 (6.9) | 5 (9.6) |
| Current smoker | Yes | 687 (21.7) | 288 (33.5) | 19 (36.5) |
| Current drinker | ≥1 time/year | 2739 (86.4) | 737 (85.8) | 42 (80.8) |
| Unhealthy eating habit | |  |  |  |
| Eating out | ≥2 times/day | 2465 (88.2) | 635 (84.9) | 46 (92.0) |
| Having breakfast | ≥3 times/week | 1629 (58.3) | 411 (54.9) | 25 (50.0) |
| Sedentary behavior (hour/day) | | 8.52±3.58 | 8.10±3.64 | 7.39±3.06 |
|  | <8 hours a day | 1205 (39.4) | 366 (45.0) | 21 (44.7) |
|  | ≥8 hours a day | 1850 (60.6) | 447 (55.0) | 26 (55.3) |
| Physical activity, MET (hr/wk) | | 30.35±39.23 | 34.35±54.27 | 43.31±71.29 |
|  | Light | 1350 (55.9) | 363 (59.8) | 15 (46.9) |
|  | Moderate to vigorous | 1065 (44.1) | 244 (40.2) | 17 (53.1) |

CVD, cardiovascular disease; BMI, body mass index; MET, metabolic equivalent.

Table 2. Prevalence of pre-diabetes and diabetes according to lifestyle variables.

| Characteristics/Variables | Category | Male (n=1,813) | | Female (n=2,377) | |
| --- | --- | --- | --- | --- | --- |
|  |  | Pre-diabetes | Diabetes | Pre-diabetes | Diabetes |
|  |  | n=503 (27.7) | n=25 (1.4) | n=387 (16.3) | n=32 (1.3) |
|  |  | n (%)^1^ | n (%) | n (%)^2^ | n (%) |
| Age (years) | 20-29 | 111 (6.1) | 3 (0.2) | 94 (4.0) | 5 (0.2) |
|  | 30-39 | 392 (21.6) | 22 (1.2) | 293 (12.3) | 27 (1.1) |
| Education | High school | 179 (10.8) | 11 (0.7) | 151 (6.7) | 13 (0.6) |
|  | ≥College | 266 (16.0) | 9 (0.5) | 219 (9.7) | 15 (0.7) |
| Marital status | Married | 303 (16.7) | 15 (0.8) | 281 (11.8) | 26 (1.1) |
|  | Unmarried | 200 (11.0) | 10 (0.6) | 105 (4.4) | 6 (0.3) |
| CVD Family history | Yes | 262 (14.8) | 17 (1.0) | 226 (9.6) | 20 (0.9) |
|  | No | 236 (13.3) | 7 (0.4) | 156 (6.6) | 11 (0.5) |
| Current job | Yes | 388 (23.3) | 18 (1.1) | 189 (8.4) | 15 (0.7) |
|  | No | 58 (3.5) | 2 (0.1) | 181 (8.0) | 13 (0.6) |
| BMI (㎏/㎡) | <25 | 221 (12.2) | 6 (0.3) | 252 (10.6) | 9 (0.4) |
|  | ≥25 | 278 (15.4) | 19 (1.1) | 135 (5.7) | 23 (1.0) |
| Perceived stress | Yes | 37 (2.1) | 1 (0.1) | 22 (0.9) | 4 (0.2) |
|  | No | 444 (25.3) | 21 (1.2) | 356 (15.3) | 26 (1.1) |
| Current smoker | Yes | 253 (14.4) | 14 (0.8) | 35 (1.5) | 5 (0.2) |
|  | Ex- or never | 228 (13.0) | 8 (0.5) | 343 (14.8) | 25 (1.1) |
| Current drinker | ≥1 time/year | 446 (25.4) | 20 (1.1) | 291 (12.5) | 22 (0.9) |
|  | Ex- or never | 35 (2.0) | 2 (0.1) | 87 (3.7) | 8 (0.3) |
| Unhealthy eating habit | |  |  |  |  |
| Eating out | ≥2 times a day | 302 (20.7) | 17 (1.2) | 333 (15.6) | 29 (1.4) |
|  | < 2 times a day | 99 (6.8) | 4 (0.3) | 14 (0.7) | 1 (0.0) |
| Having breakfast | ≥3 times a week | 206 (14.1) | 9 (0.6) | 205 (9.6) | 16 (0.7) |
|  | < 3 times a week | 195 (13.4) | 12 (0.8) | 142 (6.7) | 13 (0.6) |
| Sedentary behavior (hour/day) | <8 hours a day | 192 (11.5) | 10 (0.6) | 174 (7.7) | 11 (0.5) |
|  | ≥8 hours a day | 254 (15.3) | 10 (0.6) | 193 (8.6) | 16 (0.7) |
| Physical activity, MET (hr/wk) | Light | 183 (14.1) | 4 (0.3) | 180 (10.3) | 11 (0.6) |
|  | Moderate to vigorous | 141 (10.9) | 7 (0.5) | 103 (5.9) | 10 (0.6) |

CVD, cardiovascular disease; BMI, body mass index; MET, metabolic equivalent.

^1^Divided by the total number of male.

^2^Divided by the total number of female.

Table 3. Differences in subject characteristics and lifestyle variables between normal and prediabetic groups.

| Characteristics/Variables | Category | Male (n=1,788) | | | Female (n=2,345) | | |
| --- | --- | --- | --- | --- | --- | --- | --- |
|  |  | Normal (n=1,285) | Pre-diabetes (n=503) | *p*¹ | Normal (n=1,958) | Pre-diabetes (n=387) | *p*¹ |
|  |  | n (%) or M±SD | n (%) or M±SD |  | n (%) or M±SD | n (%) or M±SD |  |
| Age (years) |  | 29.67±5.76 | 32.90±5.00 | <0.001 | 30.30±5.82 | 32.75±5.24 | <0.001 |
| Education | High school | 544 (45.4) | 179 (40.2) | 0.060 | 656 (35.3) | 151 (40.8) | 0.045 |
|  | ≥College | 654 (54.6) | 266 (59.8) |  | 1201 (64.7) | 219 (59.2) |  |
| Marital status | Married | 515 (40.1) | 303 (60.2) | <0.001 | 1084 (55.4) | 281 (72.6) | <0.001 |
|  | Unmarried | 770 (59.9) | 200 (39.8) |  | 874 (44.6) | 105 (27.1) |  |
| CVD Family history | Yes | 551 (43.9) | 262 (52.6) | <0.001 | 1005 (51.9) | 226 (59.2) | 0.009 |
|  | No | 703 (56.1) | 236 (47.4) |  | 931 (48.1) | 156 (40.8) |  |
| Current job | Yes | 899 (74.9) | 388 (87.0) | <0.001 | 1057 (56.9) | 189 (51.1) | 0.041 |
|  | No | 302 (25.1) | 58 (13.0) |  | 802 (43.1) | 181 (48.9) |  |
| Monthly household income (10,000 Won) |  | 454.25±287.52 | 433.41±253.25 | 0.133 | 458.12±279.97 | 439.12±265.57 | 0.220 |
| BMI (㎏/㎡) |  | 23.94±3.47 | 25.85±3.71 | <0.001 | 21.69±3.144 | 24.00±4.48 | <0.001 |
| Perceived stress | Yes | 62 (4.9) | 37 (7.7) | 0.027 | 131 (6.8) | 22 (5.8) | 0.472 |
|  | No | 1191 (95.1) | 444 (92.3) |  | 1787 (93.2) | 356 (94.2) |  |
| Current smoker | Yes | 554 (44.2) | 253 (52.6) | 0.002 | 133 (6.9) | 35 (9.3) | 0.113 |
|  | Ex- or never | 699 (55.8) | 228 (47.4) |  | 1784 (93.1) | 343 (90.7) |  |
| Current drinker | ≥1 time/year | 1171 (93.5) | 446 (92.7) | 0.586 | 1568 (81.8) | 291 (77.0) | 0.031 |
|  | Ex- or never | 82 (6.5) | 35 (7.3) |  | 350 (18.2) | 87 (23.0) |  |
| Unhealthy eating habit |  |  |  |  |  |  |  |
| Eating out | ≥2 times a day | 806 (77.7) | 302 (75.3) | 0.329 | 1659 (94.3) | 333 (96.0) | 0.214 |
|  | < 2 times a day | 231 (22.3) | 99 (24.7) |  | 100 (5.7) | 14 (4.0) |  |
| Having breakfast | ≥3 times a week | 581 (56.0) | 206 (51.4) | 0.112 | 1048 (59.6) | 205(59.1) | 0.862 |
|  | < 3 times a week | 456 (44.0) | 195 (48.6) |  | 711 (40.4) | 142 (40.9) |  |
| Sedentary behavior  (hour/day) | <8 hours a day | 444 (37.1) | 192 (43.0) | 0.027 | 761 (41.0) | 174 (47.4) | 0.022 |
|  | ≥8 hours a day | 753 (62.9) | 254 (57.0) |  | 1097 (59.0) | 193 (52.6) |  |
| Physical activity, MET  (hr/wk) | Light | 446 (46.3) | 183 (56.5) | 0.002 | 904 (62.3) | 180 (63.6) | 0.669 |
|  | Moderate to vigorous | 517 (53.7) | 141 (43.5) |  | 548 (37.7) | 103 (36.4) |  |

CVD, cardiovascular disease; BMI, body mass index; METs-h per week, MET, metabolic equivalent.

¹Calculated by t-test or chi-square test.

Table 4. Differences in subject characteristics and lifestyle variables between normal and diabetic groups.

| Characteristics/Variables | Category | Male (n=1,310) | | | Female (n=1,990) | | |
| --- | --- | --- | --- | --- | --- | --- | --- |
|  |  | Normal (n=1,285) | Diabetes (n=25) | *p*¹ | Normal (n=1,958) | Diabetes (n=32) | *p*¹ |
|  |  | n (%) or M±SD | n (%) or M±SD |  | n (%) or M±SD | n (%) or M±SD |  |
| Age (years) |  | 29.67±5.76 | 34.24±4.22 | <0.001 | 30.30±5.82 | 34.38±4.89 | <0.001 |
| Education | High school | 544 (45.4) | 11 (55.0) | 0.393 | 656 (35.3) | 13 (46.4) | 0.223 |
|  | ≥College | 654 (54.6) | 9 (45.0) |  | 1201 (64.7) | 15 (53.6) |  |
| Marital status | Married | 515 (40.1) | 15 (60.0) | 0.044 | 1084 (55.4) | 26 (81.2) | 0.003 |
|  | Unmarried | 770 (59.9) | 10 (40.0) |  | 874 (44.6) | 6 (18.8) |  |
| CVD Family history | Yes | 551 (43.9) | 17 (70.8) | 0.009 | 1005 (51.9) | 20 (64.5) | 0.163 |
|  | No | 703 (56.1) | 7 (29.2) |  | 931 (48.1) | 11 (35.5) |  |
| Current job | Yes | 899 (74.9) | 18 (90.0) | 0.189 | 1057 (56.9) | 15 (53.6) | 0.727 |
|  | No | 302 (25.1) | 2 (10.0) |  | 802 (43.1) | 13 (46.4) |  |
| Monthly household income (10,000 Won) |  | 454.25±287.52 | 458.12±279.97 | 0.157 | 458.12±279.97 | 378.82±214.68 | 0.111 |
| BMI (㎏/㎡) |  | 23.94±3.47 | 27.94±5.01 | 0.001 | 21.69±3.14 | 28.25±5.19 | <0.001 |
| Perceived stress | Yes | 1191 (95.1) | 21 (95.5) | 1.00 | 1787 (93.2) | 26 (86.7) | 0.150 |
|  | No | 62 (4.9) | 1 (4.5) |  | 131 (6.8) | 4 (13.3) |  |
| Current smoker | Yes | 554 (44.2) | 14 (63.6) | 0.069 | 133 (6.9) | 5 (16.7) | 0.056 |
|  | Ex- or never | 699 (55.8) | 8 (36.4) |  | 1784 (93.1) | 25 (83.3) |  |
| Current drinker | ≥1 time/year | 1171 (93.5) | 20 (90.9) | 0.652 | 1568 (81.8) | 22 (73.3) | 0.237 |
|  | Ex- or never | 82 (6.5) | 2 (9.1) |  | 350 (18.2) | 8 (26.7) |  |
| Unhealthy eating habit | |  |  |  |  |  |  |
| Eating out | ≥2 times a day | 231 (22.3) | 4 (19.0) | 1.000 | 100 (5.7) | 1(3.3) | 0.404 |
|  | < 2 times a day | 806 (77.7) | 17 (81.0) |  | 1659 (94.3) | 29(96.7) |  |
| Having breakfast | ≥3 times a week | 581 (56.0) | 9 (42.9) | 0.23 | 1048 (59.6) | 16 (55.2) | 0.632 |
|  | < 3 times a week | 456 (44.0) | 12 (57.1) |  | 711 (40.4) | 13 (44.8) |  |
| Sedentary behavior  (hour/day) | <8 hours a day | 444 (37.1) | 10 (50.0) | 0.237 | 761 (41.0) | 11 (40.7) | 1.000 |
|  | ≥8 hours a day | 753 (62.9) | 10 (50.0) |  | 1097 (59.0) | 16 (59.3) |  |
| Physical activity, MET  (hr/wk) | Light | 446 (46.3) | 4 (36.4) | 0.560 | 904 (62.3) | 11 (52.4) | 0.371 |
|  | Moderate to vigorous | 517 (53.7) | 7 (63.6) |  | 548 (37.7) | 10 (47.6) |  |

CVD, cardiovascular disease; BMI, body mass index; MET, metabolic equivalent.

¹Calculated by t-test or chi-square test.

Table 5. Comparison of lifestyle predictors affecting prediabetes and diabetes group compared to normal group by gender

|  | Variables | Categories | Men |  |  | Women |  |  |
| --- | --- | --- | --- | --- | --- | --- | --- | --- |
|  |  |  | aOR | 95% CI | *p* | aOR | 95% CI | *p* |
| Normal *vs.* Pre-diabetes | Age (year) |  | 1.1 | 1.07-1.14 | *<.001* | 1.08 | 1.04-1.11 | *<.001* |
|  | Education | High school |  | - |  | ref |  |  |
|  |  | ≥College |  |  |  | 0.77 | 0.60-0.99 | *0.045* |
|  | Marital status | Unmarried | ref |  |  | ref |  |  |
|  |  | Married | 0.99 | 0.69-1.43 | *0.965* | 1.04 | 0.73-1.49 | *0.831* |
|  | CVD Family history | No | ref |  |  | ref |  |  |
|  |  | Yes | 1.13 | 0.86-1.49 | *0.396* | 1.12 | 0.88-1.42 | *0.376* |
|  |  |  |  |  |  |  |  |  |
|  | Current job | Yes | ref |  |  | ref |  |  |
|  |  | No | 1.02 | 0.67-1.55 | *0.934* | 1.09 | 0.85-1.39 | *0.508* |
|  | BMI (㎏/㎡) |  | 1.17 | 1.11-1.21 | *<.001* | 1.16 | 1.12-1.20 | *<.001* |
|  | Perceived stress | No | ref |  |  |  | - |  |
|  |  | Yes | 1.26 | 0.71-2.21 | *0.429* |  |  |  |
|  | Current smoker | Ex- or never | ref |  |  |  | - |  |
|  |  | Yes | 1.28 | 0.97-1.69 | 0.085 |  |  |  |
|  | Drinking | Ex- or never |  |  |  | ref |  |  |
|  |  | Current drinker |  |  |  | 0.82 | 0.62-1.09 | *0.166* |
|  | Sedentary behavior | <8 hours a day | Ref |  |  | ref |  |  |
|  | (hour/day) | ≥8 hours a day | 0.78 | 0.58-1.04 | 0.087 | 0.91 | 0.72-1.17 | *0.472* |
|  | Physical activity | Moderate to vigorous | ref |  |  |  | - |  |
|  | (MET) | Light | 1.55 | 1.17-2.05 | 0.002 |  |  |  |
| Normal *vs.* | Age (year) |  | 1.22 | 1.07-1.41 | 0.004 | 1.16 | 1.06-1.26 | 0.001 |
| Diabetes | Marital status | Unmarried | ref |  |  | ref |  |  |
|  |  | Married | 1.44 | 0.45-4.56 | 0.537 | 0.972 | 0.30-3.18 | 0.962 |
|  | CVD Family history | No | ref |  |  |  | - |  |
|  |  | Yes | 2.45 | 0.98-6.13 | 0.056 |  |  |  |
|  | BMI (㎏/㎡) |  | 1.28 | 1.15-1.42 | *<.001* | 1.43 | 1.31-1.55 | *<.001* |

aOR, adjusted odds ratio; CI, confidence interval; BMI, body mass index; CVD, cardiovascular disease.

Adjustment of normal vs. prediabetes in men: age, marital status, CVD family history, current job, BMI, stress, smoking, sedentary behavior, physical activity.

Adjustment of normal vs. diabetes in men: age, marital status, CVD family history, BMI.

Adjustment of normal vs. prediabetes in women: age, education, marital status, CVD family history, current job, BMI, drinking, sedentary behavior.

Adjustment of normal vs. diabetes in women: age, marital status, BMI.


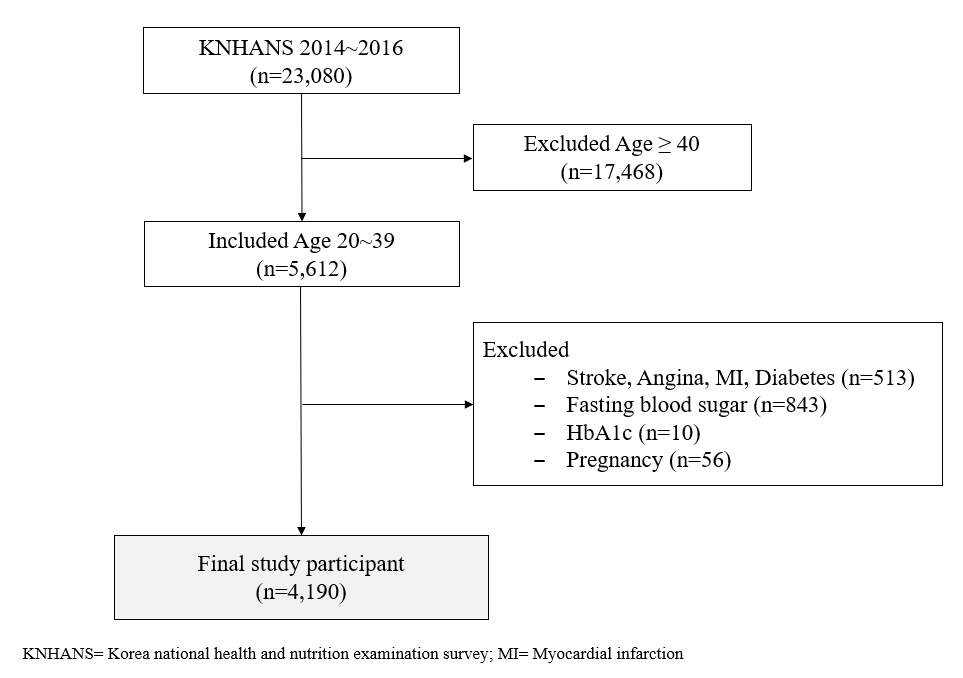


Figure 1. Flow chart of the study sample.
